# Propagation of measurement accuracy to biomass soft-sensor estimation and control quality

**DOI:** 10.1007/s00216-016-9711-9

**Published:** 2016-07-04

**Authors:** Valentin Steinwandter, Thomas Zahel, Patrick Sagmeister, Christoph Herwig

**Affiliations:** 1 Exputec GmbH, Pfeilgasse 32/20, Vienna, Austria; 2 Institute of Chemical Engineering, Research Area Biochemical Engineering, Vienna University of Technology, Gumpendorferstrasse 1a, Vienna, Austria; 3 CD Laboratory on Mechanistic and Physiological Methods for Improved Bioprocesses, Vienna University of Technology, Gumpendorferstrasse 1a, Vienna, Austria

**Keywords:** Bioprocess, Biomass estimation, Soft-sensor, Accuracy, Error propagation, Bioprocess control

## Abstract

In biopharmaceutical process development and manufacturing, the online measurement of biomass and derived specific turnover rates is a central task to physiologically monitor and control the process. However, hard-type sensors such as dielectric spectroscopy, broth fluorescence, or permittivity measurement harbor various disadvantages. Therefore, soft-sensors, which use measurements of the off-gas stream and substrate feed to reconcile turnover rates and provide an online estimate of the biomass formation, are smart alternatives. For the reconciliation procedure, mass and energy balances are used together with accuracy estimations of measured conversion rates, which were so far arbitrarily chosen and static over the entire process. In this contribution, we present a novel strategy within the soft-sensor framework (named adaptive soft-sensor) to propagate uncertainties from measurements to conversion rates and demonstrate the benefits: For industrially relevant conditions, hereby the error of the resulting estimated biomass formation rate and specific substrate consumption rate could be decreased by 43 and 64 %, respectively, compared to traditional soft-sensor approaches. Moreover, we present a generic workflow to determine the required raw signal accuracy to obtain predefined accuracies of soft-sensor estimations. Thereby, appropriate measurement devices and maintenance intervals can be selected. Furthermore, using this workflow, we demonstrate that the estimation accuracy of the soft-sensor can be additionally and substantially increased.

**Electronic supplementary material:**

The online version of this article (doi:10.1007/s00216-016-9711-9) contains supplementary material, which is available to authorized users.

## Introduction

Biotechnological process development, analysis, and control is key to obtain robust processes providing highest product quality attributes as well as a reduced time-to-market latency. Catalyzed by regulatory initiatives for biopharmaceutical products, process analytical technology (PAT) emerged as a major tool that demands for bioprocess analysis and control by frequently measurements ensuring specified final product quality [1]. Especially in biopharmaceutical production and process development of heterologous protein expression, the physiological state of the cells is highly related to the formation of critical quality attributes [2, 3]. Therefore, time-resolved knowledge about physiological parameters, such as the specific growth rate or specific substrate uptake rate, is essential in the PAT framework as well as to perform process development, characterization, and validation [4]. Moreover, those variables frequently serve as targets for control strategies [5–7]. The key to this physiological information is the catalyst concentration—the biomass. However, the required online measurement of biomass is a critical endeavor using hard-type sensors such as dielectric spectroscopy, broth fluorescence or permittivity measurements, each connected to limitations and drawbacks as outlined elsewhere [8]. Software sensors, or short soft-sensors, provide an elegant, non-invasive way to estimate biomass concentration using different other, easy-accessible measurements [9].

In this contribution, we want to focus on a dominant biotechnological process mode, the microbial fed-batch fermentation, and on the improvement of one of the most mature soft-sensor implementations using off-gas and substrate-feed measurements. These soft-sensors are established tools for bioprocess control and analysis, which was also frequently shown in practical applications [5, 10, 11]. Briefly, mass conservation laws are used to calculate turnover rates from online measurements, which might be superimposed with signal errors. In a second step accuracy of turnover rates and constraints, formulated as first-order principles such as mass and energy conservation laws, are used to reconcile the inaccurate turnover rates in order to optimally obey the constraints. Finally, the reconciled turnover rates are used to calculate the biomass formation rate (*r*_*X*_), which leads after simple integration over time to the biomass concentration. The resulting information can be used to calculate specific turnover rates, such as the specific substrate uptake rate (*q*_*S*_), which frequently serves as a control variable [12]. Therefore, *r*_*X*_ and *q*_*S*_ are regarded as the most prevailing benchmark entities to evaluate biomass estimation—and physiological control—capability.

However, the control quality by soft-sensors is limited by measurement errors of raw signals used to derive the measured turnover rates. When it comes to industrial applicability, the ultimate question is: Which measurement accuracy is required in order to obtain a sufficiently accurate estimation of the reconciled rates and the biomass?

This question can only be answered if the error sources, their respective impact, and possible counteractions are understood. We note that we use the definition of errors as deviations to the true values, excellently defined elsewhere [13]. Random errors leading to a lack of signal precision are caused by small changes within the system, e.g., air movement, temperature, and electrostatic fluctuations. A multitude of algorithms exists to smooth signals with random errors ranging from simple median filters to polynomial filters such as Savitzky-Golay filter up to frequency filters such as the Butterworth filter.

While random errors can be minimized quite easily, this is not the case for systematic errors caused by miscalibrations, inaccuracy of analytical devices, or a defective feature in the sensor. Those systematic errors can only be detected and possibly reduced by making use of all available information in terms of first-principle constraints and the accuracy of turnover rates in reconciliation procedures as outlined above. First-order principles can be generically formulated for defined processes, whereas the accuracy of turnover rates, which are input to the reconciliation procedure, are not known *a priori*. They highly depend on the accuracy of the raw signal measurements and dynamically change over time. Previously, this was approximated by propagating the variance of measurement accuracies to the turnover rates [19]. However, commonly, the expected or maximal error on measurement signals is provided by device manufacturers. Therefore, it is an existing unmet need to establish a methodology that leverages this accuracy information of the raw signals onto the derived turnover rates, which are subsequently used in the reconciliation procedure.

Hence, it is the goal of this contribution to develop an error propagation procedure to derive the accuracy of turnover rates from expected measurement errors and demonstrate its benefits in terms of increased physiological accuracy within the soft-sensor framework in microbial fed-batch mode. Moreover, in those previously elaborated methodologies, the impact of measurement error on softsensor accuracies could only be estimated retrospectively, given the process data. Therefore, we want to address the question raised above, and present a novel generic workflow that identifies tolerable measurement errors of combinations of multiple analytical measurements in order to meet the desired accuracy of soft-sensor estimations prior to conducted experiments using mechanistic knowledge.

## Material and methods

### Aim and relevance of the presented approach

The following study was carried out with in silico generated data. The aim of the in silico data generation was to obtain representative microbial fed-batch fermentation data including an induction phase, the predominant industrial mode for the production of recombinant proteins. The experiments were based on a typical *Escherichia coli* process with oxidative growth and glucose as substrate. As the batch phase is not part of the discussed soft-sensor, only the fed-batch part was considered here.

**Fig. 1:**
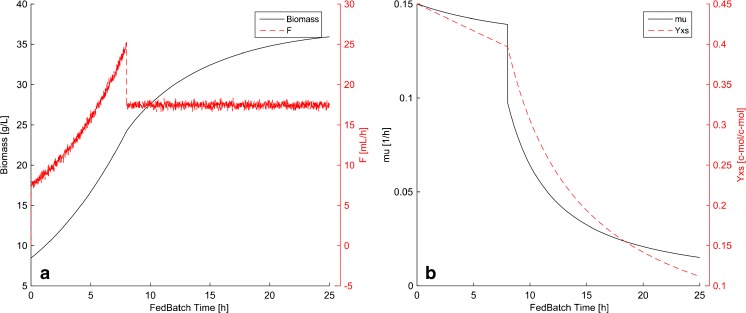
**a** Simulated feed profile and biomass concentration. **b** Simulated trajectories of the biomass/substrate yield (*Y*_X/S_) and the specific growth rate (μ)

As commonly used in industry, the modeled fed-batch phase started with an exponential feeding profile. After 8 h, the induction phase started with a linear feed rate. The biomass yield coefficient is dynamic. Due to the metabolic load, typically an especially strong decrease can be observed in the induction phase [14]. This can be measured by the soft-sensor and was also considered in the data generation process (Fig. 1).

The advantages of an in silico study are obvious:

- It is possible to “run” a bioprocess completely without any errors on the signals and to introduce defined errors into the system. This is not possible with real data, as the exact “real” values without errors on the data cannot be determined.
- A virtually infinite number of experiments with different combinations of errors can be carried out. This enables a systematic study of errors in a high-dimensional “uncertainty space.”

### Computational environment

All calculations were conducted in a MATLAB environment (2015a, The MathWorks, Inc.). The mechanistic model was created in form of a system of ordinary differential equations. As graphical user interface and bioprocessing toolbox inCyght (2016.02, Exputec GmbH) was used.

### In silico data generation

#### Main mechanistic assumptions

The main mechanistic assumptions behind data generation and soft-sensor are the same. Substrate, ammonia, and oxygen are converted to biomass and carbon dioxide. In this simple case, the extracellular formation of product or metabolites will be neglected. This assumption is true for many biopharmaceutical processes, as the product formation rate often is several order of magnitudes lower than the biomass formation rate [15]. For processes were this assumption has to be rejected, the soft-sensor framework has to be extended by online product measurement, e.g., by using spectroscopic techniques [16].

$$ {r}_S{\mathrm{CH}}_{\mathrm{pH}}{\mathrm{O}}_{\mathrm{pO}}+{r}_{\mathrm{O}2}{\mathrm{O}}_2+{r}_N{\mathrm{N}\mathrm{H}}_3\ \to {r}_X{\mathrm{CH}}_{zH}{\mathrm{O}}_{zO}{\mathrm{N}}_{zn}+{r}_{\mathrm{CO}2}{\mathrm{CO}}_2 $$

Two first principle assumptions were made; the carbon balance:

$$ {r}_S + {r}_X + {r}_{C{O}_2}=0 $$

And the degree of reduction balance:

$$ {r}_S{\gamma}_S + {r}_X{\gamma}_X + {r}_{O_2}{\gamma}_{O_2}=0 $$

The detailed list of equations for the data generation step is shown in the Electronic Supplementary Material (ESM) Section 1.

#### Addition of noise and errors on the data

To test the original and new soft-sensors with erroneous data, both systematic as well as random errors were introduced into the model. Based on information of off-gas sensor and mass flow controller manufacturers, as summarized in Table 1, realistic amounts of systematic errors were superimposed to the off-gas data which were used as input for the soft-sensors.

**Table 1:** Typical measurement errors of off-gas analyzers and mass flow controllers

	Relative error to measurement value	Measurement accuracy (zero deviance)	Drift/year
*ΔF*_*a*,in_ (mass flow controller)	±0.5 % of readout	±0.3–1 % of full scale	±1 % of full scale
$\varDelta {y}_{{\mathrm{CO}}_2,\mathrm{out}}$ (infrared)	n.a.	±1 % of full scale	±1 % of full scale
$\varDelta {y}_{{\mathrm{O}}_2,\mathrm{in}}$ (paramagnetic)	±3 % of readout	±0.2 % full scale	±2 % value
$\varDelta {y}_{{\mathrm{O}}_2,\mathrm{in}}$ (Galvanic cell)	±3 % of readout	±0.2 % full scale	±2 % value

O_2_ and CO_2_ concentrations in the exhaust gas are simply applied on the model output for the off-gas data:

$$ \begin{array}{l}{X}_{{\mathrm{O}}_2,\ \mathrm{measured}}={X}_{{\mathrm{O}}_2,\mathrm{model}}\cdot \left(1+{\upvarepsilon}_{{\mathrm{O}}_2}\right)\kern1em \\ {}{X}_{{\mathrm{CO}}_2,\ \mathrm{measured}}={X}_{{\mathrm{CO}}_2,\mathrm{model}}\cdot \left(1+{\upvarepsilon}_{{\mathrm{CO}}_2}\right)\kern1em \end{array} $$

As the error on the mass flow controller affects both total oxygen and carbon dioxide input into the system and the resulting final concentrations of O_2_ and CO_2_, the error has to be given as input to the model. The set-points for the MFC are the known values, but the model input and real values are calculated as follows.

$$ \begin{array}{l}{F}_{{\mathrm{O}}_2,\mathrm{in},\mathrm{model}}=\frac{F_{{\mathrm{O}}_2,\mathrm{in},\mathrm{setpoint}}}{1+{\upvarepsilon}_{MFC}}\hfill \\ {}{F}_{{\mathrm{CO}}_2,\mathrm{in},\mathrm{model}}=\frac{F_{{\mathrm{O}}_2,\mathrm{in},\mathrm{setpoint}}}{1+{\upvarepsilon}_{\mathrm{MFC}}}\hfill \end{array} $$

For the errors in the feed rate, a relative error on the set-point rate is applied.

$$ {r}_{S,\mathrm{model}} = \frac{r_{S,\mathrm{setpoint}}}{1+{\upvarepsilon}_{r_S}} $$

The amounts of systematic errors applied for the different experiments are listed in “Comparison of soft-sensor estimates to unbiased model data” section.

The model delivered an online value each seven seconds. For the addition of random error, white Gaussian noise was added to the off-gas signals. The noise was generated by using MATLAB’s awgn function with a relative standard deviation of 1 % for the off-gas data and 10 % on the feed rate. The noise on the feed rate is typically relatively high, as the signal often is calculated by deriving the scale signal.

### Quantitative evaluation of bioprocess data and error propagation

#### Preprocessing

As described in the “Introduction” section, random errors can be minimized by using preprocessing methods. We decided to apply a Savitzky-Golay filter with a window size of 30 min and second-degree polygon on the off-gas signals. These parameters in most cases showed a low signal distortion, while on the other hand, the elimination of noise was good. However, it has to be noted that for specific filtering and smoothing problems, better filters and filter parameters may exist. In our experience, most of them are not generically applicable, meaning that if they work very well for a specific problem on a defined signal with specific signal dynamics, they may completely fail on another.

#### Data-driven rate calculation

The aim of the next section is to express estimators for those conversion rates derived from measurements. In general, all conversion rates can be formulated using the simple idea, that the conversion rate equals the net accumulation within the reactor minus the inflow into the reactor plus the outflow out of the reactor.

For demonstration purpose of the subsequent error propagation, the calculation of the conversion rate for CO_2_ will be shown exemplarily:

$$ {r}_{{\mathrm{CO}}_2}\kern0.5em =\kern0.5em \mathrm{C}\mathrm{E}\mathrm{R}\kern0.5em =\kern0.5em \frac{d\left({\mathrm{CO}}_2\right)}{dt\ }\kern0.5em -\dot{{\mathrm{CO}}_{2,\mathrm{in}}}\kern0.5em +\kern0.5em \dot{{\mathrm{CO}}_{2,\mathrm{out}}} $$

The term $\frac{d\left({\mathrm{CO}}_2\right)}{dt\ }$ can be neglected since it is predominantly a function of pH and temperature, which were kept constant over all in silico simulations. Therefore, the carbon emission rate (CER) formulates to:

$$ \mathrm{C}\mathrm{E}\mathrm{R}\kern0.5em =\kern0.5em \frac{F_{a,\mathrm{in}}}{V_m}\left({y}_{{\mathrm{CO}}_2,\mathrm{out}}\cdot R{a}_{\mathrm{inert}}-{y}_{{\mathrm{CO}}_2,\mathrm{in}}\right) $$

Where *Ra*_inert_ is the inert gas ratio, which connects the inflow to the outflow by:

$$ R{a}_{\mathrm{inert}}=\frac{F_{a,\mathrm{out}}}{F_{a,\mathrm{in}}} $$

And is defined as:

$$ R{a}_{\mathrm{inert}}\kern0.5em =\kern0.5em \frac{1-{y}_{{\mathrm{O}}_2,\mathrm{in}}-{y}_{{\mathrm{CO}}_2,\mathrm{in}}}{y_{{\mathrm{O}}_2,\mathrm{out}}-{y}_{{\mathrm{CO}}_2,\mathrm{out}}-\frac{y_{\mathrm{wet}}}{y_{{\mathrm{O}}_2,\mathrm{in}}}} $$

Here, *y*_wet_ is the oxygen concentration in the off-gas stream without bio-reaction and indirectly relates to water stripping out of the reactor.

Well-known procedures can be applied in order to calculate the substrate- and oxygen- uptake rate for a substrate limited *E. coli* fermentation as described elsewhere [15].

#### Error propagation

In general, all the input signals for estimating the conversion rates are random variables, associated with a random and systematic error, therefore the estimators itself are random variables, too. As discussed in the “Introduction” section, random errors in the raw signals can be minimized using preprocessing methods, whereas systematic errors cannot be removed and propagate directly to the estimated conversion rates. However, via Gaussian error propagation, it is possible to estimate the expected error of the conversion rates. This knowledge will subsequently help us to formulate a much more robust reconciliation procedure and estimation of biomass.

The influence of the absolute measurement error (*Δy*) of the signal *y* onto a derived signal *r* can be approximated using a Taylor expansion [3]:

$$ r\left(y+\varDelta y\right)=r(y) + \frac{1}{1!}\frac{dr(y)}{dy}\cdotp \varDelta y+\frac{1}{2!}\frac{d{}^2r(y)}{dy{}^2}\cdotp {\left(\varDelta y\right)}^2+\dots $$

We want to note that the absolute measurement error *Δy* of the measurement signal can be in most cases calculated from technical device data sheets given by their maximal amplitude (e.g., ±3 % of readout). Therefore, the absolute measurement error *Δy* can be seen as worst-case error. For an approximate solution, the Taylor expansion can be terminated after the second term and the resulting absolute deviation of the derived signal (*Δr*) can be written as:

$$ r\left(y+\varDelta y\right)-r(y) = \varDelta r=\frac{dr(y)}{dy}\cdotp \varDelta y $$

If the derived signal (here the conversion rate) depends on more than one input variable and the error of the input signal is only known by its boundaries, which is the typical case for biotechnological applications, we can write in analogy:

$$ \varDelta r=\left|\frac{\partial r}{\partial {y}_1}\right|\cdotp \varDelta {y}_1+\left|\frac{\partial r}{\partial {y}_2}\right|\cdotp \varDelta {y}_2+\dots $$

For the CER, the error propagation formulates to:

$$ \begin{array}{l}\varDelta \mathrm{C}\mathrm{E}\mathrm{R}=\left|\frac{\partial \mathrm{C}\mathrm{E}\mathrm{R}}{\partial {F}_{a,\mathrm{in}}}\right|\cdot \varDelta {F}_{a,\mathrm{in}}+\left|\frac{\partial \mathrm{C}\mathrm{E}\mathrm{R}}{\partial {V}_m}\right|\cdot \varDelta {V}_m+\left|\frac{\partial \mathrm{C}\mathrm{E}\mathrm{R}}{\partial {y}_{{\mathrm{CO}}_2,\mathrm{out}}}\right|\cdot \varDelta {y}_{{\mathrm{CO}}_2,\mathrm{out}}+\left|\frac{\partial \mathrm{C}\mathrm{E}\mathrm{R}}{\partial R{a}_{\mathrm{inert}}}\right|\cdot \varDelta R{a}_{\mathrm{inert}}+\left|\frac{\partial \mathrm{C}\mathrm{E}\mathrm{R}}{\partial {y}_{{\mathrm{CO}}_2,\mathrm{in}}}\right|\cdot \varDelta {y}_{{\mathrm{CO}}_2,\mathrm{in}}\hfill \\ {}\varDelta \mathrm{C}\mathrm{E}\mathrm{R}=\left|\frac{1}{V_m}\left({y}_{{\mathrm{CO}}_2,\mathrm{out}}\cdot R{a}_{\mathrm{inert}}-{y}_{{\mathrm{CO}}_2,\mathrm{in}}\right)\right|\cdot \varDelta {F}_{a,\mathrm{in}}+\left|\frac{F_{a,\mathrm{in}}}{V_m^2}\left({y}_{{\mathrm{CO}}_2,\mathrm{out}}\cdot R{a}_{\mathrm{inert}}-{y}_{{\mathrm{CO}}_2,\mathrm{in}}\right)\right|\cdot \varDelta {V}_m+\left|\frac{F_{a,\mathrm{in}}}{V_m}\cdot R{a}_{\mathrm{inert}}\right|\cdot \varDelta {y}_{{\mathrm{CO}}_2,\mathrm{out}} + \left|\frac{F_{a,\mathrm{in}}}{V_m}\cdot {y}_{{\mathrm{CO}}_2,\mathrm{out}}\right|\cdot \varDelta R{a}_{\mathrm{inert}}+\left|\frac{F_{a,\mathrm{in}}}{V_m}\right|\cdot \varDelta {y}_{{\mathrm{CO}}_2,\mathrm{in}}\hfill \\ {}\varDelta {R}_{a,\mathrm{in}\mathrm{ert}}=\left|\frac{1-{y}_{{\mathrm{O}}_2,\mathrm{in}}-{y}_{{\mathrm{CO}}_2,\mathrm{in}}}{\left({y}_{{\mathrm{O}}_2,\mathrm{out}}-{y}_{{\mathrm{CO}}_2,\mathrm{out}}-\frac{y_{\mathrm{wet}}}{y_{{\mathrm{O}}_2,\mathrm{in}}}\right){}^2}\right|\cdot \varDelta {y}_{{\mathrm{O}}_2,\mathrm{out}}+\left|\frac{1-{y}_{{\mathrm{O}}_2,\mathrm{in}}-{y}_{{\mathrm{CO}}_2,\mathrm{in}}}{{\left({y}_{{\mathrm{O}}_2,\mathrm{out}}-{y}_{{\mathrm{CO}}_2,\mathrm{out}}-\frac{y_{\mathrm{wet}}}{y_{{\mathrm{O}}_2,\mathrm{in}}}\right)}^2}\right|\cdot \varDelta {y}_{{\mathrm{CO}}_2,\mathrm{out}}+\left|\frac{1-{y}_{{\mathrm{O}}_2,\mathrm{in}}-{y}_{{\mathrm{CO}}_2,\mathrm{in}}}{\frac{1}{y_{{\mathrm{O}}_2,\mathrm{in}}}\cdot \left({y}_{{\mathrm{O}}_2,\mathrm{out}}-{y}_{{\mathrm{CO}}_2,\mathrm{out}}-\frac{y_{\mathrm{wet}}}{y_{{\mathrm{O}}_2,\mathrm{in}}}\right){}^2}\right|\cdot \varDelta {y}_{\mathrm{wet}}\hfill \end{array} $$

**Fig. 2:**
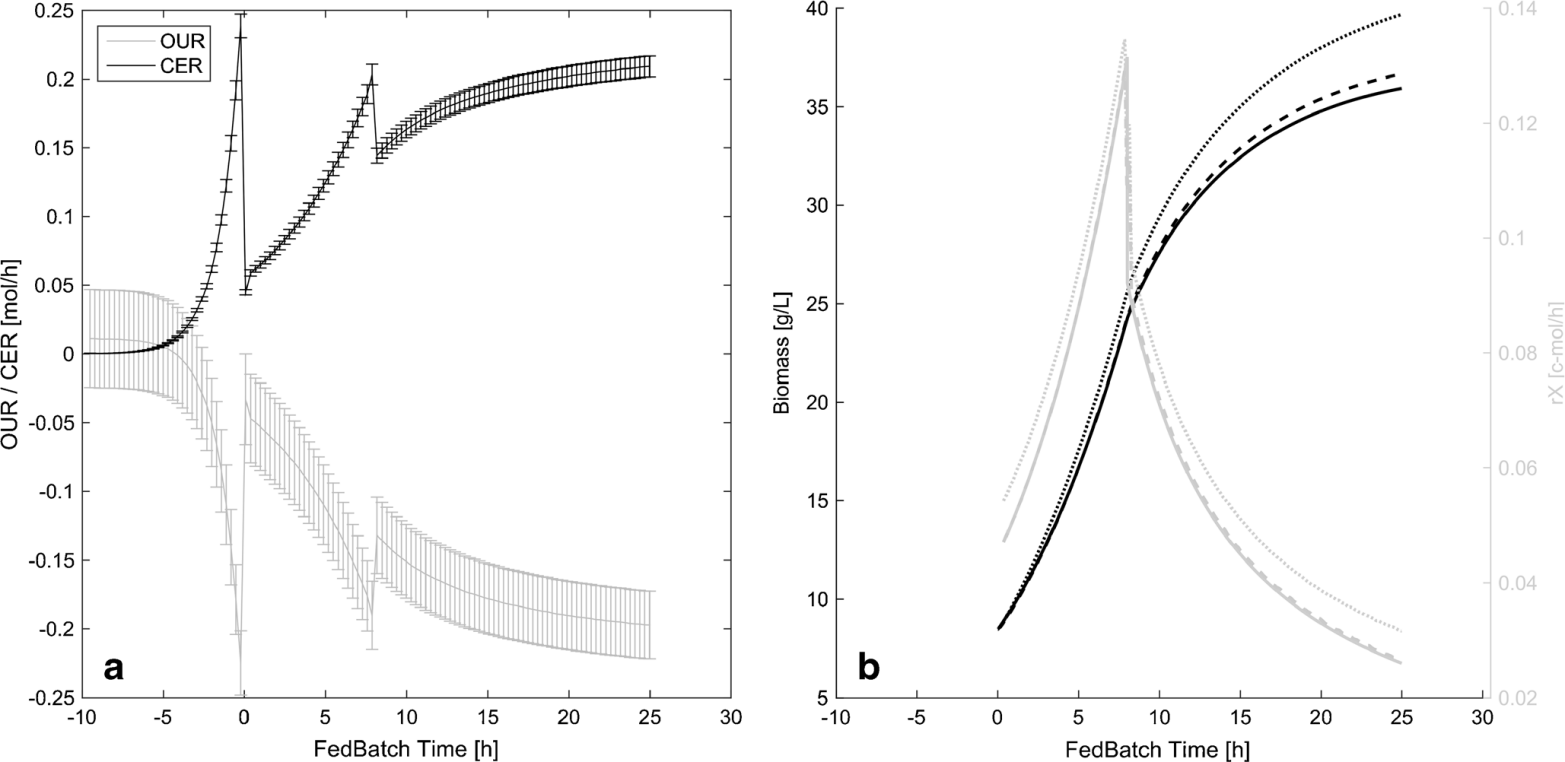
**a** Time-resolved profiles for OUR and CER are shown together with their respective accuracy as error *bars*, calculated by error propagation as described in “Error propagation” section. **b** Comparison of biomass (*black*) and *r*_*x*_ (*gray*) soft-sensor prediction to the unbiased signals (*solid lines*). Estimations of traditional soft-sensor implementations, assuming 3 % error of all input rates, are shown *dotted*; the adapted soft-sensor with error propagation for the input rates is shown in *dashed lines*. For this particular simulation, raw signals were superimposed by 2 % error for CO_2_ and O_2_ off-gas concentration, respectively, and 1 % error on the *r*_*S*_ and the MFC, respectively

This procedure can easily be extended to the OUR and the substrate uptake rate (*r*_*S*_). Typical results of error propagation to the off-gas rates are shown in Fig. 2a.

For the presented in silico study input signals: *F*_*a*,in_, ${y}_{{\mathrm{CO}}_2,\mathrm{out}}$ for CER and ${y}_{{\mathrm{O}}_2,\mathrm{out}}$ for the OUR, were regarded as superimposed with considerably relevant systematic measurement error. All other input signals were considered to be perfectly accurate. Typical errors for mass flow controllers and off-gas analytics are given in Table 1. However, the error propagation model could be easily extended to more inputs with systematic error. For the error propagation of *r*_*S*_, the only considerably source of systematic signal error was the concentration of the substrate, which might vary due to evaporation during sterilization procedures.

#### Minimum variance rate reconciliation and biomass soft-sensor estimation

In the following section, we want to briefly summarize an established minimum variance reconciliation and biomass estimation procedure in order to reduce systematic error on measured turnover rates (OUR, CER, and *r*_*S*_) using first principles as reported in detail elsewhere [15, 17, 18].

First principles, such as elemental balances (see “Main mechanistic assumptions” section), can be seen as constraints to the bioreactor system. We can formulate many of those constraints and thereby connect components with each other. Commonly, a compact matrix formulation is used to connect conversion rates of components with each other using multiple constraints:

$$ E\cdot r=0 $$

*E* is the elemental composition matrix [*e* × *n*] with *e* being the number of elemental balances and *n* the number of relevant components. *r* is the vector containing the turnover rates. Under real conditions, the elemental balances do not close due to systematic errors of the rates with a residual vector *ε*:

$$ E\cdot r=\varepsilon $$

For this in silico example, the elemental *C* balance as well as the degree of reduction (DoR) balance were used as frequently applied elsewhere [5].

In order to identify gross errors in the system, it is necessary to check if the residual vector (*ε*) differs significantly from zero. Therefore, Reilly and Carpani introduced a statistical measure (*h* value) which weights the residuals by their accuracy (covariance matrix). Using this *χ* ^2^ distributed measure, it is possible to set confidence levels for detecting gross errors [18].

The presented concept can easily be extended to more elemental balances or energy balances; however, the implementation with C- and DoR-balance is predominantly implemented in industry since additional measurements (e.g., nitrogen or heat transfer) are more complex to realize in practice.

The redundancy of the measurable rates (rank of redundancy matrix R [15, 17, 18]) equals 1, and therefore this redundancy can be used to balance the measured rates in a minimum variance sense and obtain reconciled rates [15].

After no gross error could be detected with statistical significance and the measured rates are reconciled, those reconciled rates can be finally used to estimate the biomass formation rate, which is the only non-measured rate in this example. For this minimum variance balancing procedure, the covariance matrix of the measured signals is required. A fair assumption is to state that the covariance of the measured rates is diagonal, which assumes non-correlated errors in the measured signals. In current soft-sensor implementations, an empirical approach was chosen and the covariance of all measured rates was assumed to equal 3 % of the readout [10, 15]. As a unique feature of the presented soft-sensor implementation, we will use at this point the derived error boundaries from above as worst-case estimators for the variances of the signals. Since the herein-derived error boundaries vary dynamically over time, the new approach will be further on called adaptive soft-sensor.

### Comparison of soft-sensor estimates to unbiased model data

As a methodology to investigate the result of the soft-sensor as a function of the error of the input signals, we investigated 5915 in silico experiments with systematically varied errors on the off-gas measurements, substrate concentration, and mass flow controlled (described in “Addition of noise and errors on the data” section). The selected ranges in Table 2 were based on technical manufacturer information of MFC and off-gas analyzer (see Table 1).

**Table 2:** All errors listed here were combined with each other and applied in 5915 experiments. All those in silico generated data sets were used to test the prediction accuracy of traditional and adaptive soft-sensor approach

	Applied relative error on in- and outputs	Step size
${\varepsilon}_{{\mathrm{CO}}_2,\kern0.75em }{\varepsilon}_{{\mathrm{CO}}_2}$	−3 to +3 %	0.5 %
${\varepsilon}_{r_S}$	−3 to +3 %	1 %
*ε*_MFC_	−2 to +2 %	1 %

As a final output of the soft-sensor, the estimated biomass formation rate (${\hat{r}}_X$) was compared to the true, unbiased biomass formation rate from the in silico model (*r*_*X*,*true*_), which is known. This comparison was done by calculating the median percentage of difference (MPD) over all data points of the time series according to:

$$ \mathrm{M}\mathrm{P}\mathrm{D}\kern0.75em =\kern0.5em 100\cdot \mathrm{median}\left(\frac{{\hat{r}}_X-{r}_{X,\mathrm{true}}}{r_{X,\mathrm{true}}}\right) $$

**Fig. 3:**
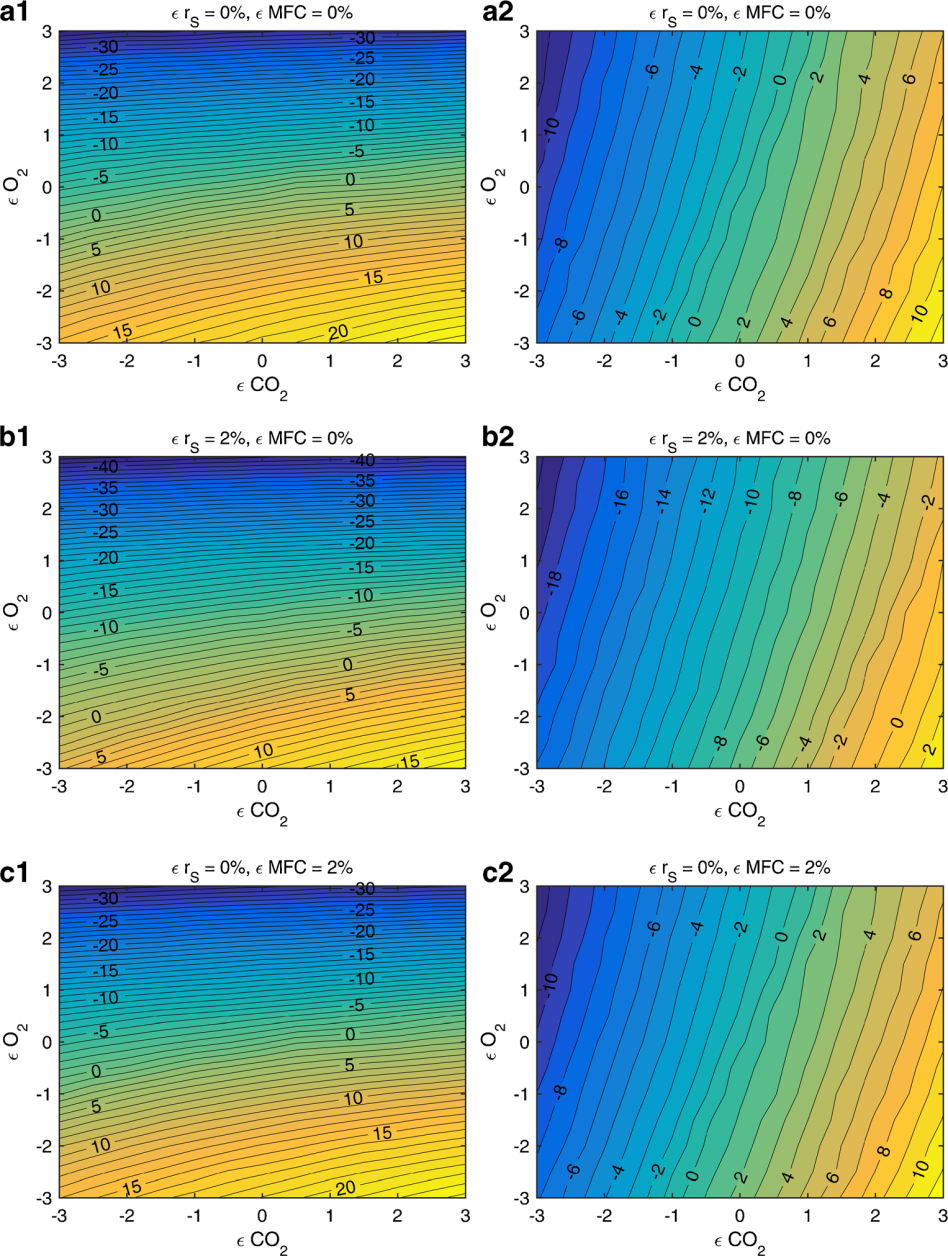
Comparison of *r*_*X*_ for traditional (*left*) and adapted (*right*) soft-sensor in terms of MPD, showing the deviation between the real biomass formation rate and the soft-sensor values (%) as a function of the errors on the off-gas data. (*a*) Errors on the off-gas data, but no errors on r_S_ and the MFC. (*b*) Errors on the off-gas data and 2 % error on *r*_*S*_. (*c*) Errors on the off-gas data and 2 % error on the MFC

**Fig. 4:**
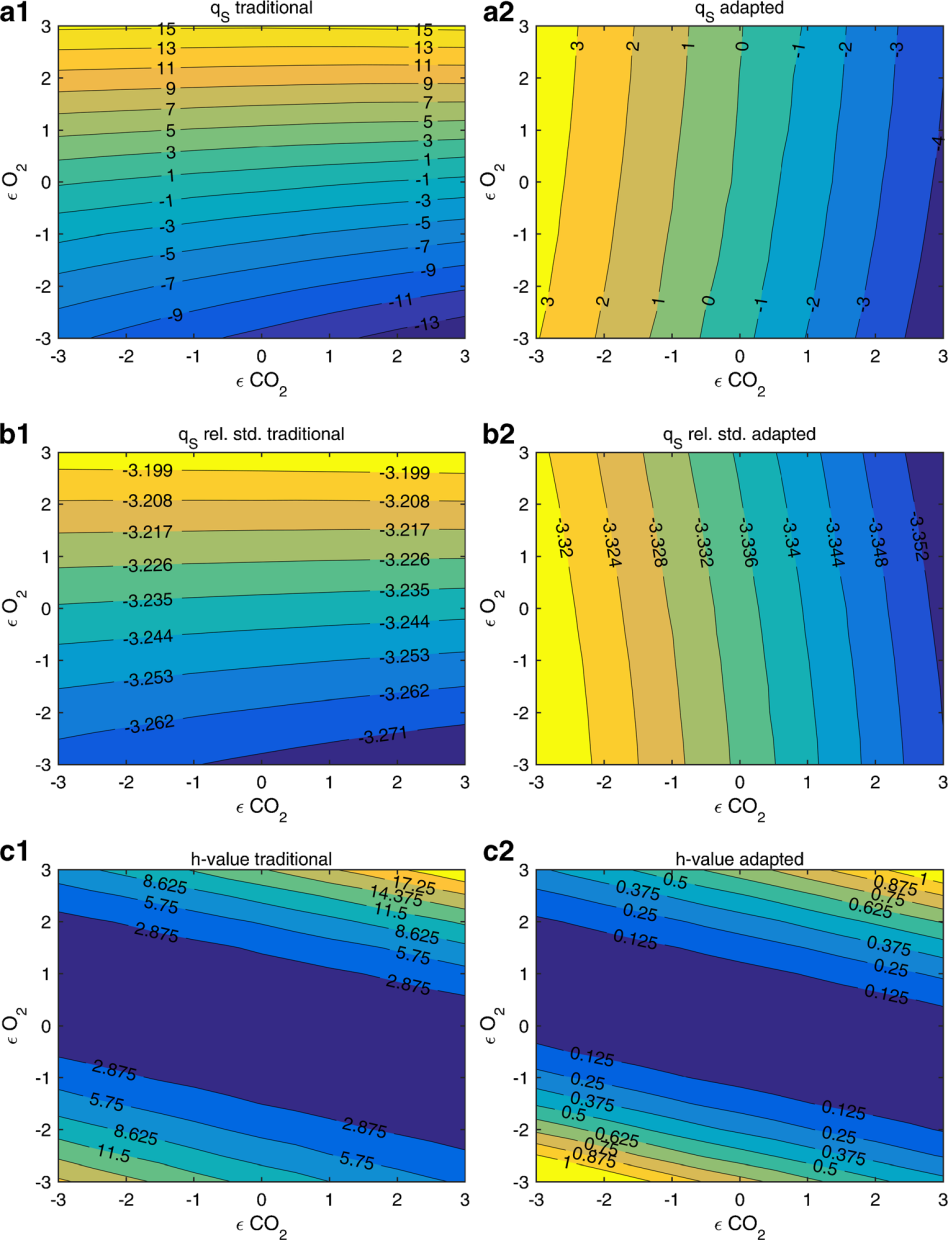
Comparison of *q*_*S*_ in terms of MPD (*a*), the median of the estimated relative standard deviation on the reconciled *q*_*S*_ (*b*), and the median *h* values (*c*) for traditional (*left*) and adapted (*right*) soft-sensor, depending on the error level of the off-gas analyzers

For each of the 5915 simulations, a MPD value for *r*_*X*_ and *q*_*S*_ was calculated. Those values are displayed as surface plots as shown in Figs. 3 and 4.

## Results

### Comparison of the soft-sensors accuracy

#### Biomass formation rate

In the following sections, a comparison between the traditional approach and the adaptive soft-sensor is done. While traditionally, the errors on CER, OUR, and *r*_*S*_ were estimated to be 3 % for all rates and over the whole process, the adapted soft-sensor calculates the accuracy on the rates through error propagation, by making use of the known uncertainty ranges of the raw signals. The herein dynamically resolved accuracy for OUR and CER, assuming 3 % maximal error on the read out of off-gas analytical measurements of O_2_ and CO_2_, are depicted in Fig. 2a. The accuracy of CER is much higher than the accuracy of OUR. This information is used by the adapted soft-sensor; therefore, we obtain much more accurate biomass and *r*_*X*_ estimates than previous implementations without error propagation, compared to unbiased biomass and *r*_*X*_ signals, as shown in Fig. 2b.

Since Fig. 2b shows only the results for one particular error combination of errors on O_2_, CO_2_, *r*_*S*_ and the MFC, we have to resolve the predictions with all other error combinations in order to show superiority of the adapted soft-sensor. Figure 3 shows a comparison of the biomass formation rate (*r*_*X*_) between the true rates (model) and the estimated rates (left column: traditional soft-sensor, right column: adaptive soft-sensor) by means of MPD. In each of the subfigures, the MPD is shown as a function of the error on the O_2_ and CO_2_ off-gas concentration, varied between −3 and +3 %. The error on the MFC and on *r*_*S*_ was varied across the rows of the subfigures.

When using the traditional approach (subplots on the left side), especially errors on the oxygen signal lead to high errors on the estimated rates as well as much higher MPD values (up to 42 % compared to maximal 19 % for the adaptive soft-sensor) and almost horizontal lines of equivalent MPD lines in Fig. 3. The adaptive soft-sensor propagates the measurement accuracy to the turnover rates, which makes the OUR less trustworthy than the CER as indicated by the error bars in Fig. 2. Therefore, also errors on the CO_2_ off-gas measurement have an impact on the MPD values, resulting in a rotation of the equivalent MPD lines to the diagonal direction (subplots on the right of Fig. 3). This change of influential parameters on the MPD will be observed multiple times throughout this work and is always caused by changing the accuracy of turnover rates to realistic values using the error propagation procedure.

In the subplots (a1) and (a2) in Fig. 3, the MPD values regarding *r*_*X*_ are shown as a function solely of error on O_2_ and CO_2_ off-gas measurement. In the subplots b1 and b2, we added a relative error of 2 % on *r*_*S*_ to the true model values and in subplots c1 and c2, we added an error of 2 % on the MFC set-point. The average MPD values as well as the maximal MPD values (up to 40 vs. 18 %) reached throughout all subplots are much lower for the adaptive soft-sensor compared to the traditional approach.

#### Control quality for specific substrate uptake rate

As one of the main applications of the soft-sensor is the process control based on physiological parameters, the two versions of the soft-sensors were also compared in terms of prediction accuracy for the specific substrate uptake rate *q*_*S*_.

Here, subplots (a1) and (a2) in Fig. 4 show the MPD for *q*_*S*_ with varying error on O_2_ and CO_2_ measurement and no error on *r*_*S*_ and MFC signal. Again, the adaptive soft-sensor shows on average much lower MPD values as well as much lower maximal MPD values (up to 15 % for the traditional and up to 4 % for the adaptive soft-sensor). b1 and b2 show that the estimated relative standard deviation for both soft-sensors is in the area of 3 %. However, when looking at the results in A1 and A2, only the adaptive soft-sensor delivers the estimated standard deviations, since MPD values are in the range of ±3 %.

As shown in Fig. 4(c1 and c2), the *h* values of the traditional soft-sensor quickly exceed levels of 3. In this case, the null hypothesis, that there is no gross error in the system, has to be rejected with a confidence level of 95 %. However, the system had no gross error in reality, and as the *h* values of the adaptive soft-sensor show, the null hypothesis cannot be rejected when using the correctly calculated covariance matrix for the minimum variance reconciliation. Therefore, the *h* values of the traditional soft-sensor have no statistical significance as the covariance matrix, as explained before, is not correctly estimated.

#### Integrated comparison over the entire uncertainty space

The goal of this section is to derive a global parameter which we can use to judge which soft-sensor approach leads to generally more accurate estimations. In general, we face a four-dimensional input space consisting of different errors on the O_2_, CO_2_, *r*_*S*_, and MFC measurement. This space will be subsequently called the uncertainty space. At each point in this uncertainty space, the MPD of the true model value of *r*_*X*_ and *q*_*S*_ is compared to the two soft-sensor approaches. Taking the mean of all those MPD values of the uncertainty space for each soft-sensor approach gives us a clear measure which soft-sensor implementation is generally more accurate. This integrated parameter will be called the global average MPD.

Table 3 summarizes the results of this kind of analysis and shows for each cell the mean MPD of simulations where the O_2_ and CO_2_ error was varied between −3 and +3 %, analogous to one subplot of Fig. 3. In the columns of Table 2, the error on the MFC is varied, in the rows the error on *r*_*S*_. The global average MPD regarding *r*_*X*_ of the adaptive soft-sensor is 8.7 % compared to 15.2 % of the traditional approach. This is a reduction of the MPD by 43 %. For the estimation of the specific substrate uptake rate *q*_*S*_, the global average MPD can be even lowered from 7.6 to 2.7 which corresponds to a MPD reduction of 64 %.

**Table 3:**
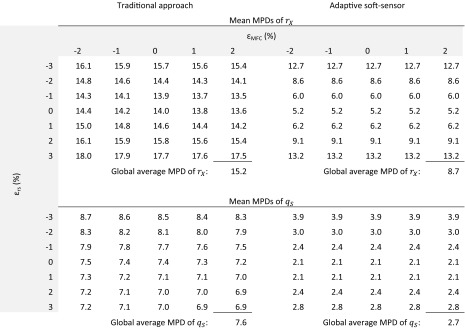
Comparison of traditional and adaptive soft-sensor for different error levels. Each of the cells show the mean MPD value of simulations in which the error on O_2_ and CO_2_ was varied between −3 and +3 %

### Generic workflow to ensure appropriate control quality

**Fig 5:**
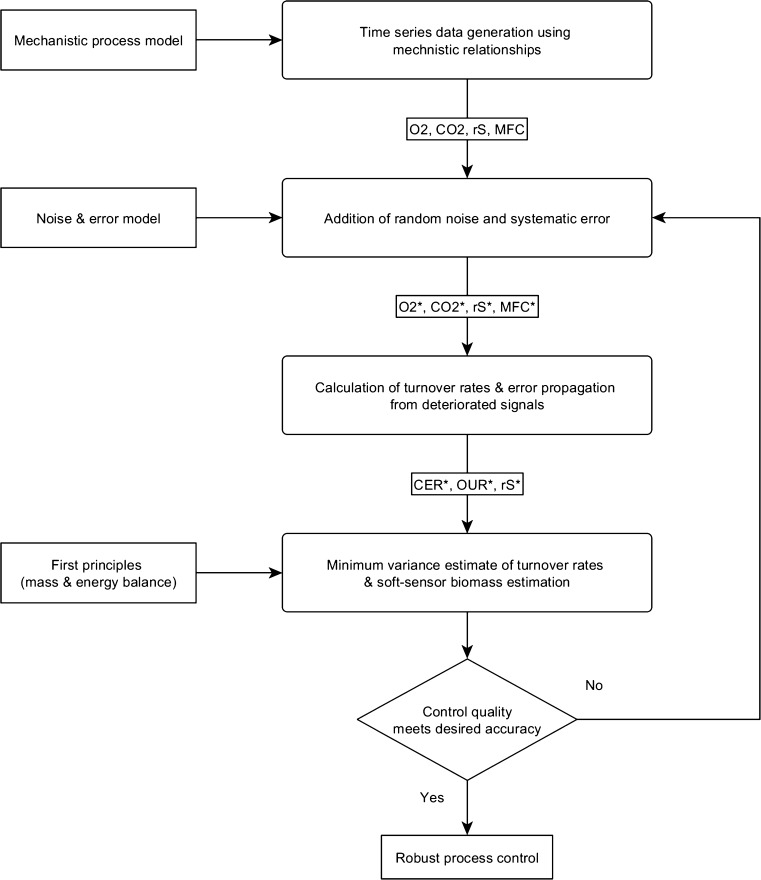
Generic workflow for identification of desired measurement error and noise for robust biomass soft-sensor estimation. *Asterisk* indicate variables which were superimposed by random noise and systematic error

Besides showing superior behavior of the new soft-sensor implementation over state of the art, we want to present a novel generic workflow to obtain a desired soft-sensor estimate or reconciliation quality by adapting accuracy of measurement devices. This workflow includes the following steps as indicated in Fig. 5:

1. Use a mechanistic process model to generate time-resolved data which will be used to derive rates. These are used as input to the soft-sensor (here O_2_ and CO_2_ concentration of the off-gas stream, substrate concentration and inflowing air controlled by MFC).
2. Obtain biased signals by superimposing them with representative systematic (see manufacturer specifications) and random noise (estimated process noise).
3. Calculate turnover rates including their accuracy as described in “Data-driven rate calculation” section. The rates and their accuracy (covariance) are input to the soft-sensor.
4. Use the soft-sensor’s first principles to reconcile measured turnover rates unless gross errors are detected. The reconciled rates can be used to estimate the biomass and all related entities (e.g., *q*_*S*_ or biomass).
5. The herein obtained *q*_*S*_ estimate (or biomass estimate) is compared to the true, unbiased model signal. If the estimate does not meet the predefined thresholds (e.g., 5 % global average MPD), more accurate measurement devices and their respective measurement errors are used to continue with step 2 to 5 with reduced systematic error levels. The selection of appropriate measurement devices is driven by technical, manufacturing, and financial constraints, which is not scope of this study.
6. If the estimate meets the predefined thresholds in terms of global average MPD, a robust estimate under industrial relevant process conditions is achieved.

**Fig. 6:**
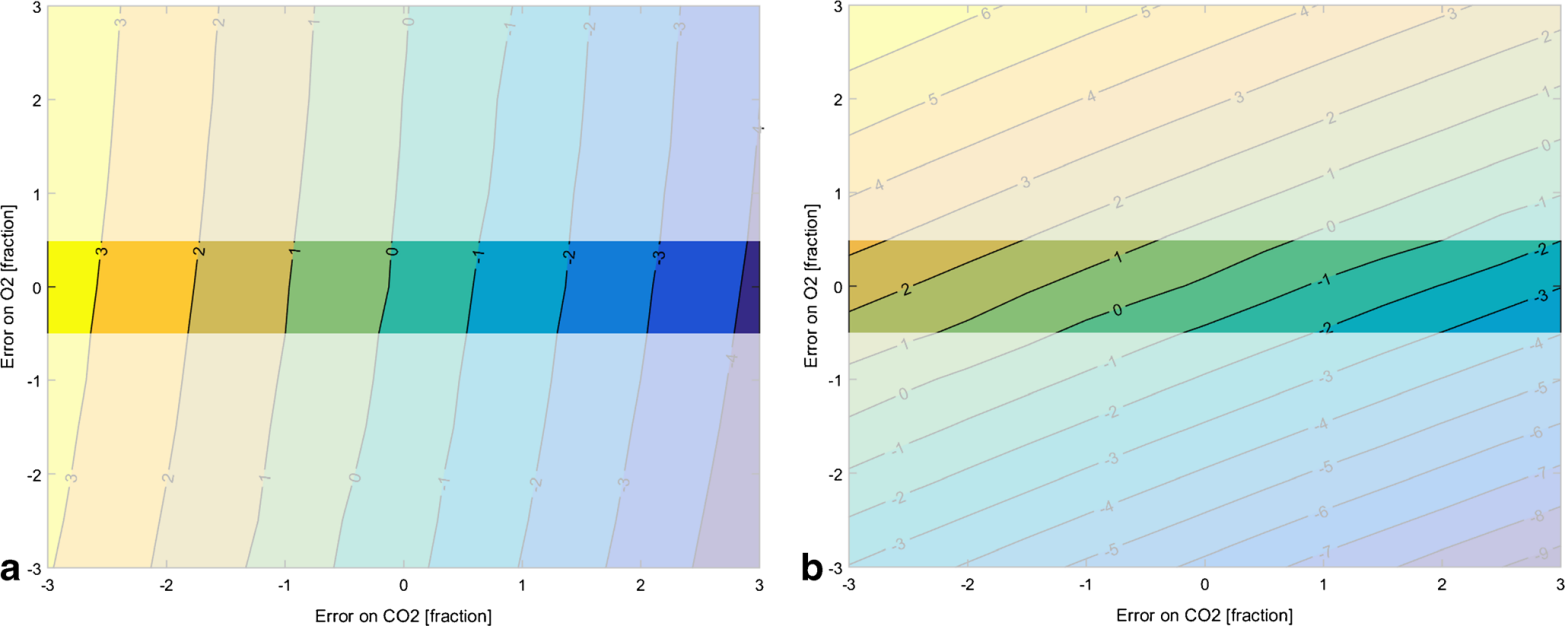
Estimation error of *q*_*S*_ before (**a** ±3 % maximal error on oxygen measurement and ±2 % maximal error on MFC) and after (**b** ±0.5 % maximal error on oxygen measurement and ±1 % maximal error on MFC) the increase of the input signal accuracies. Not only values outside the uncertainty range can be excluded but also the mean MPD inside the uncertainty space is more accurate

As an example, the error ranges of Table 2 were taken as a starting position in step 2 of the generic workflow presented in Fig. 5. It was assumed that the desired control quality could not be reached with the current analytical devices (step 5), therefore, exemplary a higher accuracy of the oxygen sensor and MFC from ±3 to ±0.5 % and ±2 to ±1 %, respectively, was implemented. The results before and after this change are shown in Fig. 6. After the change, the estimated error surface of *q*_*S*_ is rotated in a favorable direction to enlarge regions of lower error (0 to 2 % error), as depicted in the non-shaded areas of the two subfigures of Fig. 6. Overall, this results in 10 % reduced global average MPD.

## Discussion

### Superior accuracy for the estimated rates of the adaptive soft-sensor

As perceived in Figs. 2, 3, and 4(a1 and a2) as well as summarized in Table 1, the adaptive soft-sensor delivers more accurate estimates of *r*_*X*_, which integrates to biomass, and *q*_*S*_. Moreover, maximal MPD values for *r*_*X*_ and *q*_*S*_ are much lower for the adaptive soft-sensor which implies that the biomass estimate as well as the control of *q*_*S*_ can be performed much more robust under real process conditions since large deviations to the true values of *r*_*X*_ and *q*_*S*_ can be avoided.

This is due to the fact that the covariance matrix for the minimum variance reconciliation procedure is arbitrary chosen for the traditional soft-sensor, which assumes a too low uncertainty range for the OUR, as shown exemplarily in Fig. 2. The adaptive soft-sensor on the other hand dynamically uses all available information (uncertainty ranges of off-gas analysis) to calculate a realistic covariance matrix. This leads to a much more robust estimate of *r*_*X*_ and *q*_*S*_.

For each subfigure, there are some “sweet spots” for certain error combinations, where the classical soft-sensor shows a better accuracy in the prediction of *r*_*X*_. Since the exact combination of the present errors on the input signals is not known *a priori*, this is no advantage under real process conditions.

In a previous approach, the uncertainty of turnover rates was approximated by propagation of variance [19]. However, under industrial applications, the maximal expected error is provided or known as an empirical parameter. Moreover, in the previous work, simulations were not used to systematically investigate the true error obtained by softsensor estimations. Therefore, our approach including the generic workflow offers the possibility to pre-access the expected dynamics of the process and influence of measurement errors on soft-sensor predictions using mechanistic modeling.

### Statistical meaningfulness of standard deviation and *h* value

The covariance matrix is a critical input for the minimum variance reconciliation procedure. As for the traditional soft-sensor, the covariance matrix consists of arbitrary values which do not represent the true and dynamically changing uncertainties; all calculated statistical measures, i.e., standard deviation and *h* value, lose their significance. This is different for the adaptive soft-sensor.

As shown in Fig. 4(b1 and b2), the relative standard deviations of the estimated rates are in the range of 3 % and almost identical for traditional and adaptive soft-sensor. However, when comparing these standard deviations to the actually measured errors in terms of MPD in Fig. 4(a1 and a2), it becomes clear that the calculated standard deviations do not fit to these errors for the traditional soft-sensor; the measured MPDs go up to 15 % in the considered area. For the adaptive soft-sensor, the standard deviations are meaningful and on the same magnitude as the actually measured MPDs. Under real process conditions, the calculated standard deviation of an estimated rate is the only available measure to evaluate their prediction accuracy and expected uncertainty and is therefore of critical importance.

As shown in Fig. 4(c1 and c2), the *h* values of the traditional soft-sensor quickly exceed levels of 3. As already explained in “Control quality for specific substrate uptake rate” section, the calculated *h* values are statistically not meaningful. This means that they cannot be used to detect a gross error in the system with a defined level of significance. They only can be used to relatively compare similar processes or detect gross errors when the *h* values are magnitudes higher than expected. This is not true for the adaptive soft-sensor, as over the whole uncertainty space the *h* values are below 3, and no false positive detection of gross errors occurred with 95 % confidence.

### Applicability of the generic workflow to set measurement accuracies and ensure desired accuracy of soft-sensor estimations

The question about the required measurement accuracy of raw signals to meet the desired accuracy of derived variables, such as the soft-sensor estimation for bioprocess control, is equally urged by device manufacturer as by process engineers. This is due to the fact that measurement accuracy is often correlated to higher asset costs of advanced devices or more frequent maintenance intervals of existing devices.

In “Generic workflow to ensure appropriate control quality” section, we present a generic workflow to answer this question. Since the measurement accuracy of the derived soft-sensor estimate is not only a function of the accuracy of the input signals (step 2 of the workflow) but also of the dynamics of the process, a mechanistic model has to provide this information (step 1 of the workflow). If one has multiple possibilities of exchanging devices or maintenance intervals to increase accuracy of input signals, this can be solved iteratively in the workflow by testing different of those combinations and evaluating if the resulting accuracy of the soft-sensor is sufficient. Moreover, as shown in Fig. 6, it is thereby possible to not only get rid of areas with high levels of MPD but rather additionally increase the accuracy in the reduced uncertainty space due to the introduction of supplementary knowledge about the accuracy of input signals.

### Extrapolations of the adaptive soft-sensor and the generic workflow to other application areas

The presented error propagation approach as well as the presented workflow are generically applicable to include additional sources of information. For example, the consideration of energy balances based on the metabolic heat production during a process [20] or the already mentioned combination of the soft-sensor with spectroscopic techniques [16] could be included. This would result in an even more robust and diverse applicable package.

## Conclusion

In this contribution, we aim to present an error propagation procedure increasing the accuracy and robustness of the soft-sensor estimates.

Traditionally, the uncertainties for conversion rates (CER, OUR, *r*_*s*_) were arbitrarily assumed and static over the whole process. Here, we established a novel procedure to obtain meaningful uncertainties, dynamically changing over time, which are used as representative knowledge source together with first principles in the soft-sensor framework.

In this in silico case study, the new approach using the adaptive soft-sensor, the error on the estimates could be reduced by 43 % for the estimated biomass growth rate (*r*_*X*_) compared to traditional soft-sensor implementations. For the estimation of the specific substrate uptake rate *q*_*S*_, the error on the estimate could even be lowered by 64 %.

When using the traditional soft-sensor approach, the resulting *h* values could not be used to statistically reject the null hypothesis of detecting gross errors, since estimations of covariance of the turnover rates were arbitrarily chosen and static over time. The new approach delivers both statistically meaningful *h* values for the detection of gross errors and informative standard deviations on the estimated rates. Latter ones are essential under real process conditions to judge soft-sensor estimation quality, as obviously there exist no possibility to evaluate the control quality by comparing the estimates to unbiased model values.

Additionally, we presented a new generic approach to ensure a predefined control quality of the soft-sensor estimate by iteratively evaluating the effect of the different errors on the raw signal measurements. It has been demonstrated that by following this generic workflow, it is possible to additionally significantly increase the adaptive soft-sensor accuracy.

The presented approach can be generically applied taking also additional error sources into account. The new methodology is practically applicable to industrial conditions, where maximal errors of measurement devices are used to obtain dynamically changing accuracies of derived turnover rates as shown in Fig. 2a.

## Electronic supplementary material

Below is the link to the electronic supplementary material.

ESM 1 (PDF 651 kb)

## Abbreviations

CER: Carbon dioxide evolution rate (mol h^−1^)
*E*: Elemental composition matrix
*F*_*a*,in_: Air flow in (L min^−1^)
*F*_*a*,out_: Air flow out (L min^−1^)
MFC: Mass flow controller
MPD: Median percentage of difference
OUR: Oxygen uptake rate (mol h^−1^)
*q*_*S*_: Specific substrate uptake rate (mol mol^−1^ h^−1^)
*Ra*_inert_: Inert gas ratio (−)
*r*_*i*_: Consumption/formation rate for species *i* (mol h^−1^)
*r*_*X*_: Biomass formation rate (mol h^−1^)
*S*: Substrate, C-normalized (mol)
*V*_*m*_: Molar volume (L mol^−1^)
*X*: Biomass, C-normalized (mol)
*µ*: Specific growth rate (h^−1^)
*ε*: Residual vector for non-closing balances
*y*_wet_: Oxygen fraction in the off-gas without microbial activity (−)
${y}_{{\mathrm{O}}_2,\mathrm{out}}$: Oxygen fraction in the off-gas stream (−)
${y}_{{\mathrm{O}}_2,\mathrm{in}}$: Oxygen fraction in the inlet air (−)
*γ*_*i*_: Degree of reduction for species *i* (−)
${y}_{{\mathrm{CO}}_2,\mathrm{out}}$: Carbon dioxide fraction in the off-gas stream (−)
${y}_{{\mathrm{CO}}_2,\mathrm{in}}$: Carbon dioxide fraction in the inlet air (−)
*Y*_*X*/*S*_: Biomass/substrate yield coefficient
*ε*_*i*_: Applied relative error on species *i* (−)
*Δy*: Absolute measurement error of signal *y*
