# Supplementary material for: Propagation of measurement accuracy to biomass soft-sensor estimation and control quality
Source: Anal Bioanal Chem. 2016 Jul 4;409(3):693–706. doi: 10.1007/s00216-016-9711-9 (PMC5233751; doi:10.1007/s00216-016-9711-9)
Supplement: Supplementary file 1 — (PDF 651 kb) [file 216_2016_9711_MOESM1_ESM.pdf]

Analytical and Bioanalytical Chemistry

Electronic Supplementary Material

Propagation of measurement accuracy to biomass soft-sensor estimation and control quality

Valentin Steinwandter, Thomas Zahel, Patrick Sagmeister, Christoph Herwig

1 Main mechanistic assumptions and equations

The main mechanistic assumptions behind data generation and soft-sensor are the same. Substrate, ammonia and oxygen is converted to biomass and carbon dioxide. As the amounts of products in biopharmaceutical processes are in the ranges of some milligrams per liter, the formed product can be neglected.

$$r_S CH_{pH} O_{pO} + r_{O2} O_2 + r_N NH_3 \rightarrow r_X CH_{zH} O_{zO} N_{zn} + r_{CO2} CO_2$$

Main input signal into the model are the stoichiometry of the substrate ($C_6H_{12}O_6$) and the concentration (0.400 g mL^{-1}) and feed rate of the substrate. The stoichiometry of the organism was taken from literature [1].

The feed rate at time point t during the not induced fed-batch phase was calculated in form as follows. After the induction phase, the feed rate was kept constant (relevance shown in Figure 1 of the main document).

$$F(t) = F_0 * e^{\mu t}$$

t is the time (h), µ the feed exponent (h^{-1}) and F_0 the feed rate (mL) at fed-batch start which was calculated

$$F_0 = \frac{X * M_S * \mu}{Y_{X/S} * M_X * c_S}$$

Where *X* is the total amount of biomass in the reactor (g), M_X the C-normalized molecular weight of the biomass (g c-mol^{-1}), $Y_{X/S}$ the biomass substrate yield (c-mol c-mol^{-1}), c_S the feed concentration (g mL^{-1}) and M_S the C-normalized molecular weight of the substrate (g c-mol^{-1}).

As there is no substrate accumulation and no outflow of substrate during the fed-batch phase, the complete inflow of substrate is immediately consumed, resulting in a substrate uptake rate r_S (c-mol h^{-1}) which is only dependent on the feed rate and the concentration of the feed.

$$r_S = -\frac{(F * c_S)}{M_S}$$

It should be mentioned here, that all rates where the flow direction shoes into the cell or where a species is consumed, were defined to be negative (r_S, *OUR*), while rates leading to an accumulation or formation of a species were defined to be positive (r_X, *CER*).

The biomass formation rate r_X (c-mol h^{-1}) was calculated by using a fixed biomass/substrate yield in the exponential fed-batch phase, and a decreasing biomass/substrate yield in the induction phase.

$$r_X = -r_S * Y_{X/S}$$

The consumption of oxygen per formed amount of biomass $Y_{O2/X}$ (mol c-mol^{-1}) was calculated by setting up the electron balance. γ_S, γ_S and γ_{O2} are the degrees of reduction based on one c-mole of substrate and biomass (c-mol^{-1}), or one mole of oxygen (mol^{-1}), respectively. The degrees of reduction were calculated by setting up γ_N = -3, γ_C = 4, γ_H = 1 and γ_O = -2 [2]. As γ for CO_2, NH_3 and H_2O according to the previous definition is 0, the degree of reduction in the system sum should not change over time.

$$r_S\gamma_S + r_X\gamma_X + r_{O_2}\gamma_{O_2} = 0$$

When setting r_X to 1 and and r_S to $1/Y_{X/S}$, r_{O2} corresponds to $Y_{O2/X}$ and can be calculated according to the following equation:

$$Y_{O_2/X} = \frac{\frac{\gamma_S}{Y_{X/S}} - \gamma_X}{\gamma_{O_2}}$$

Using $Y_{O2/X}$, the oxygen uptake rate *OUR* (mol h^{-1}) now can be simply calculated.

$$OUR = Y_{O_2/X} * r_X$$

For the calculation of the carbon dioxide evolution rate *CER* (mol h^{-1}) it was assumed that the whole carbon flux goes into the biomass or leaves the reactor as carbon dioxide. When neglecting product formation and extracellular metabolites, the carbon balance can be stated as follows. All sum formulas are normalized to one carbon, resulting in the following equation:

$$r_S + r_X + r_{CO_2} = 0$$

As the accumulation of carbon dioxide in the reactor can be neglected, the carbon dioxide evolution rate was calculated as follows:

$$CER = r_{CO_2} = Y_{CO_2/S} * -r_S = \left(1 - Y_{X/S}\right) * -r_S$$

In the last step, the used oxygen and the produced carbon dioxide are added and subtracted from the inlet air and oxygen, considering water stripping and assuming the whole gas phase as ideal gas. The oxygen fraction in the air ($y_{O2,\,Air}$) is 0.2095, the oxygen fraction of the oxygen supply tank 0.9800 ($y_{O2,\,O2}$). The volumetric inflow of oxygen $O_{2,\,in}$ (L h^{-1}) is calculated as follows:

$$F_{O_2,in,total} = y_{O_2,Air} * F_{Air,in} + y_{O_2,O_2} * F_{O_2,in}$$

The volumetric oxygen outflow $F_{O2,\,out}$ (L h^{-1}) is

$$F_{O_2,out} = F_{O_2,in,total} + (OUR * V_M)$$

Similar for carbon dioxide $F_{CO2,\,out}$ (L h^{-1})

$$F_{CO_2,out} = F_{CO_2,in} + (CER * V_M)$$

In the final step, the total outflow is calculated and the detected values $X_{CO2,\,out}$ and $X_{O2,\,out}$ (%) are generated. The value $y_{O2,\,wet}$ represents the oxygen content of the exhaust gas without microbial activity. It is an important value to estimate the water stripping effect and described in detail elsewhere [3]. First the total outflow of air $F_{Air,\,out}$ has to be calculated.

$$F_{Air,out} = \frac{F_{Air,in,total} + CER * V_M + OUR * V_M}{y_{O_2,wet} / y_{O_2,Air}}$$

In the end, the volumetric percentage fraction of oxygen and carbon dioxide can be calculated as follows:

$$X_{O_2,out} = 100 * \frac{F_{O_2,out}}{F_{Air,out}}$$

$$X_{CO_2,out} = 100 * \frac{F_{CO_2,out}}{F_{Air,out}}$$

2 References

1. Doran PM. 4 - Material Balances [Internet]. In: Bioprocess Engineering Principles. London: Academic Press; 1995. page 51–85.Available from: http://www.sciencedirect.com/science/article/pii/B9780122208553500043

2. Villadsen J, Nielsen J, Lidén G. Bioreaction Engineering Principles [Internet]. Boston, MA: Springer US; 2011 [cited 2016 Feb 6]. Available from: http://link.springer.com/10.1007/978-1-4419-9688-6

3. Heinzle E, Oeggerli A, Dettwiler B. On-line fermentation gas analysis: Error analysis and application of mass spectrometry. Anal. Chim. Acta 1990;238:101–15.
